# Complex systems representing effective connectivity in patients with Type One diabetes mellitus

**DOI:** 10.1371/journal.pone.0208247

**Published:** 2018-11-29

**Authors:** Joan Guàrdia-Olmos, Esteve Gudayol-Ferré, Geisa B. Gallardo-Moreno, Mar Martínez-Ricart, Maribel Peró-Cebollero, Andrés A. González-Garrido

**Affiliations:** 1 Facultat de Psicologia, Universitat de Barcelona, Institut de Neurociències, Institute of Complex Systems (UBICS), Barcelona, Spain; 2 Facultad de Psicología, Universidad Michoacana de San Nicolás de Hidalgo, Francisco, Michoacán, México; 3 Instituto de Neurociencias, Universidad de Guadalajara, Guadalajara, Jalisco, México; 4 Facultat de Psicologia, Universitat de Barcelona, Barcelona, Spain; Nathan S Kline Institute, UNITED STATES

## Abstract

**Background:**

Type 1 diabetes mellitus (T1D) affects the entire cellular network of the organism. Some patients develop cognitive disturbances due to the disease, but several authors have suggested that the brain develops compensatory mechanisms to minimize or prevent neuropsychological decline. The present study aimed to assess the effective connectivity underlying visuospatial working memory performance in young adults diagnosed with T1D using neuroimaging techniques (*f*MRI).

**Methods:**

Fifteen T1D right-handed, young adults with sustained metabolic clinical stability and a control group matched by age, sex, and educational level voluntarily participated. All participants performed 2 visuospatial working memory tasks using a block design within an MRI scanner. Regions of interest and their signal values were obtained. Effective connectivity—by means of structural equations models—was evaluated for each group and task through maximum likelihood estimation, and the model with the best fit was chosen in each case.

**Results:**

Compared to the control group, the patient group showed a significant reduction in brain activity in the two estimated networks (one for each group and task). The models of effective connectivity showed greater brain connectivity in healthy individuals, as well as a more complex network. T1D patients showed a pattern of connectivity mainly involving the cerebellum and the red nucleus. In contrast, the control group showed a connectivity network predominantly involving brain areas that are typically activated while individuals are performing working memory tasks.

**Conclusion:**

Our results suggest a specific effective connectivity between the cerebellum and the red nucleus in T1D patients during working memory tasks, probably reflecting a compensatory mechanism to fulfill task demands.

## Introduction

Type 1 diabetes mellitus (T1D) is a metabolic disease that has been directly related to several clinical impairments, such as retinopathies, nephropathies, and neuropathies, along with an increased risk of cognitive decline [1]. Patients with T1D may show mild cognitive impairment [2] that usually affects memory, processing speed, verbal skills, learning, and executive functions, including working memory in both children [3, 4, 5, 6, 7] and adults [8, 9, 10, 11, 12].

Despite such alterations, adult patients with T1D may show cognitive impairment circumscribed to certain cognitive areas, with severity varying from mild to moderate or having no cognitive alterations at all [11]. Accordingly, several functional magnetic resonance (*f*MRI) studies–both in activation and brain connectivity papers—suggest that the brains of T1D patients develop several functional adaptations that prevent or limit the deleterious effect of T1D on cognitive processing. Several of those papers focus on the study of working memory.

Working memory (WM) is a cognitive function that allows us to temporarily keep and manipulate information in memory in a wide range of cognitive tasks [13]. Different meta-analyses have suggested that, for it to work properly, the activity of several cortical areas is needed; these areas include the bilateral premotor cortex (BA 6.8), the medial cingulate cortex and the supplementary motor area (BA 32.6), the frontal anterior pole (BA 10), the dorsolateral prefrontal cortex (BA 9 and 46), the ventrolateral cortex (BA 45 and 46), and the posterior parietal cortex (BA 40) [14, 15, 16]. Additionally, some extracortical areas participate in WM tasks, such as the basal ganglia [17, 18], the cerebellum [19], and the thalamus [20], and during visuospatial WM tasks, the occipitotemporal cortex and the hippocampus have also been implicated [21].

Brain activity during WM tasks has shown important differences between T1D patients and healthy controls and between T1D patients with different clinical characteristics or glycemic states. Diabetic patients with retinopathy show less deactivation in the anterior cingulate cortex and the orbitofrontal cortex in the state of hypoglycemia compared to patients without retinopathy [22]. In other work, the patients with T1D in euglycemia showed a similar activation pattern as healthy controls during WM tasks, but during a period of hypoglycemia, the diabetic patients had greater activations in the parietal and the frontal cortex, as well as in the cerebellum and the hippocampus, when performing the task [23]. All these studies were performed using classical approaches to *f*MRI analysis that are useful to study functional segregation, which is a basic principle of brain organization [24]. However, a particular area of the brain may be involved in different cognitive and behavioral functions working in coordination with other brain areas. The study of this functional integration can be conducted by functional and effective connectivity *f*MRI analyses [24]. The brain’s functional connectivity is defined as a pattern of statistical dependence between distant neurophysiological events, while effective connectivity denotes the effects that a brain area can exert over others [24]. The study of brain connectivity in the past decades during the brain’s rest allowed us to discover several functionally linked regions that form functional networks within the central nervous system, such as the motor network, the visual networks, the default mode network (DMN), and the cognitive control network, among others (see [25] for a review). Functional connectivity may also be studied during the performance of a particular cognitive or emotional task to study the functional integration that subserves these activities [26].

Brain connectivity at rest in patients with T1D may also be altered. Some studies have suggested that some children and adults with the disease will show brain hyperconnectivity patterns related to a good cognitive performance that had been interpreted as brain adaptations to prevent or limit cognitive impairment in diabetic patients [27, 28]. Additionally, abnormal connectivity patterns in adults have been reported between the bilateral subgenual cingulate cortex and other structures of the prefrontal cortex and the DMN in diabetic patients [29]. The work by Bolo et al. [23] suggested that the DMN of T1D patients was hyperactive with respect to the control group during a WM task. Adult patients with this disease show a relative lack of high-level cortical hubs in the prefrontal cortex [12, 30] and a global topology of the gray matter network with a rather random organization in adult T1D patients when compared to healthy subjects [31].

The literature suggests that patients with T1D show both a brain functional activation pattern different from that observed in healthy controls during different WM tasks, and they also show functional connectivity patterns at rest that differ from those obtained in healthy controls. Nevertheless, to our knowledge, there are no functional connectivity studies during WM tasks in this type of patient. Therefore, our goal was to evaluate brain connectivity during a visuospatial WM task in a sample of young adults with T1D, and we studied the brain activity pattern through *f*MRI related to this cognitive function [32, 33]. In these previous studies, T1D patients and the healthy control group showed activations expected by the task in areas of the lateral prefrontal cortex, anterior cingulate cortex and cerebellum, but T1D patients showed less cortical activations than the control group in the left parietal cortex, premotor cortex, and superior frontal gyrus and more intense activations than the controls in the inferior frontal gyrus, basal ganglia and cerebellum. In addition, T1D patients showed activations in the substantia nigra that were not observed in the healthy participants [32, 33].

In light of the results of our two precedent studies, our hypothesis is that the control subjects will show a pattern where brain connectivity networks will be established between the cortical areas typically related to WM, whereas the connectivity pattern of the patients with T1D will instead be focused on other brain structures such as the cerebellum and subcortical structures such as the basal ganglia. This is a very general hypothesis, but it is not feasible to formulate *a priori*, a more specific one, given that the current study has been conducted on the basis of the ROIs activated (data-driven) independently in each of the tasks, which constitutes a clearly different approach from what we carried out in our previous papers (hypothesis-driven). We believe that such differences would be due to specific mechanisms of brain adaptation and compensation that make it possible to maintain cognitive efficiency, as reflected by the analysis of effective connectivity between different brain regions. For this study, we introduced the use of structural equation models (SEM), as they have proven a high level of efficiency in the estimation of complex networks similar to those formulated here. In addition, they are in turn easy and available to estimate [26, 34].

## Materials and methods

### Participants

The study’s samples consisted of 21 subjects with T1D and 21 without T1D, selected through intentional sampling among the outpatients at the *Hospital Civil Fray Antonio Alcalde* and the *Servicio de Endocrinología del Centro Médico Nacional de Occidente*, Guadalajara, Mexico. All the participants met the inclusion criteria: i) they were right-handed, ii) they were between 14 and 30 years old, iii) they had an IQ within the normal range, and iv) they had a minimum of 9 years of school education. The group of participants with T1D consisted of subjects with a minimum 4 years since the onset of the disease, with an onset between childhood and adolescence. The control group was mostly recruited through the same subjects with T1D, and they were paired by age, gender, and years of school education.

Regarding exclusion criteria, we discarded data from subjects with i) a history of neurodevelopmental alterations; ii) signs of depression according to Hamilton’s Depression Rating Scale; iii) substance dependence or abuse; and iv) frequent periods of hypo- or hyperglycemia that had ended in one or more hospitalizations in the last two years. Later, the participants who did not finish the experimental tasks or who moved during the completion of the tasks were also removed. In the end, a total of 12 participants were excluded, thus leading to a final sample of 15 subjects with T1D and 15 subjects without T1D with strict pairings.

Table 1 shows the descriptive statistics for the sociodemographic data from both samples and the comparison between both groups. There were no differences between the two groups, with the exception of total IQ, verbal comprehension index and plasma glucose before the *f*MRI (mg/dL). However, the total IQ score or the verbal comprehension index score in the two groups show that both distributions were normogroups.

**Table 1:** Demographic and clinical characteristics of the study subjects.

	Patients with T1D	Control subjects	Signification
*n*	15	15	
Age (years)	20.6 (4.0)	21.13 (4.41)	No Sig.
Sex (men/women)	9/7	9/7	No Sig.
Education (years)	12.69 (2.87)	13.31 (2.75)	No Sig.
TOTAL IQ	103.88 (7.40)	113.06 (7.30)	*p* = 0 .04
Verbal Comprehension Index	102.88 (12.39)	116.81 (8.73)	*p* < .01
Perceptual Reasoning Index	109.19 (8.31)	113.44 (8.41)	No Sig.
Working Memory Index	97.00 (2.12)	99.50 (3.08)	No Sig.
Processing Speed Index	104.38 (16.36)	118.44 (10.87)	*p* < .01
Diabetes duration (years)	10.44 (5.37)		
HbA_1c_ (%)	8.91 (2.09)		
(mmol/mol)	74 (22.8)		
Last fasting plasma glucose (mg/dL)	128.54 (60.05)		
Plasma glucose before *f*MRI (mg/dL)	207.06 (72.31)	106.8 (40.19)	*p* < .01

The data are presented as the means (SD); n = number of cases; HbA1c = glycated hemoglobin.

### Experimental task

**Fig 1:**
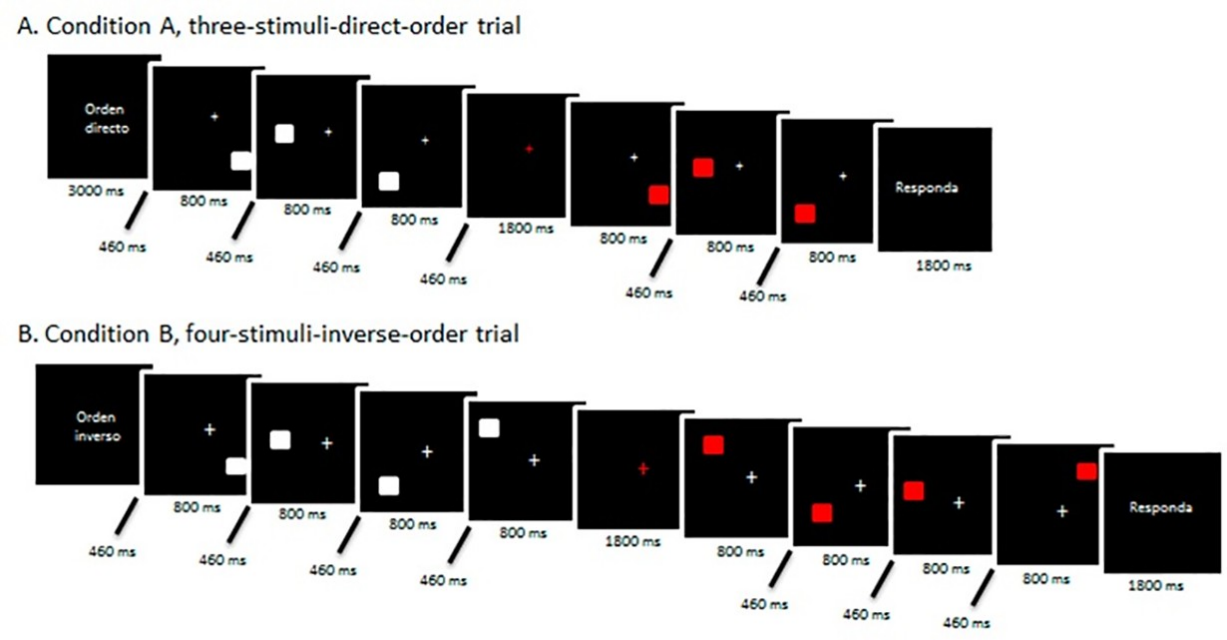
Layout of the tasks applied in the experimental sequence.

The experimental task had a block design (boxcar) and consisted of a visuospatial short-term memory task and a visuospatial working memory task (Tasks A and B, respectively). Both visuospatial memory tasks involved visualizing a square on the screen, which moved to 3 or 4 different positions. Next, after a first sequence, a second sequence was played that could be the same as or different from the previous sequence. In Task A, the participant received the instruction “DIRECT ORDER”, and upon finishing the task, they had to answer whether the order of the positions of the square in the second sequence matched the order of the positions in the first sequence. Task B had the exact same format as A, but in this case, the instruction given was “REVERSE ORDER”, and the subject had to answer whether the order of the positions of the square in the second sequence followed the exact same order as in the first, but in reverse order. Each task lasted 21 seconds and was composed of 2 blocks, one with 3 stimuli and another with 4. In each block of stimuli, two sequences were presented. The first sequence was called “sequence of visualization”, where the participant had to pay attention to the order in which the squares were presented. In the second sequence, called “sequence of response”, the squares kept, or did not, the previously presented spatial order (Fig 1). The participants had to respond by pressing button 1, if the second sequence correctly matched the first one, according to the instruction received, and button 2 if they did not.

The total time of acquisition of the images was 6:12 minutes for both tasks. The visuospatial memory paradigm comprised 16 blocks, 8 blocks of tasks for block A and block B presented alternately, and 8 blocks of rest were presented after each task block.

### Experimental procedure

At the beginning of each block, the instructions “DIRECT ORDER” or “REVERSE ORDER” were shown at the center of the screen, followed by a white fixation point (cross). Thereafter, the three or four squares, also white, were sequentially presented at different positions of the screen. The first sequence was followed by the reappearance of the white cross, the fixation point, that preceded the presentation of the second sequence of squares–this time printed in red–matching or not the spatial order in which the first sequence had been presented. To conclude, the instruction “Respond” appeared on the screen, and the subject was given time to answer. The initial instruction had a duration of 3,000 ms. The presentation of each stimulus lasted 800 ms, and the interstimulus interval lasted 460 ms. For the period between sequences and the answer time, both lasted 1,800 ms each.

Image acquisition: The experimental tasks were programmed with E-prime Studio v.2.0 (Psychology Software Tools, Inc., 2010) and were projected through a goggle system. The answers were registered in a four-button box compatible with a magnetic resonance. For the image acquisition, a 1.5 Tesla GE ExcieHDxT scanner (General Electric Medical System, Milwaukee, WI) was used with an eight-channel standard circular antenna with headpads to restrict head movement. The first scan was a locator for the positioning of the planes of the functional image. Previous to the study, T2 axial series were obtained for every subject in addition to a sequence pulse of rapid three-dimensional high-resolution images (SPGR) to be used as anatomic references. For the functional images, 32 cuts were obtained and acquired in ascending sequential order, with a 4-mm thickness with no space between them, in an oblique axial plane, by using eco-planar images (EPI), with a time of repetition (TR) of 3,000 ms, a time of echo (TE) of 60 ms, and a flip-angle of 90° in a 64x64 matrix. As a result, the voxel size was 4.06 x 4.06 x 4 mm.

### General procedure

The study’s protocol was approved by the ethics committee of the *Hospital Civil Fray Antonio Alcalde*, and all the persons taking part received ample explanation on the reason and the details of the study. Likewise, all their doubts were answered before signing the written informed consent. In the case of minors, consent was obtained from the parents. In the first contact with the patients, the reason for the study was explained, and those who were willing to participate were administered a brief questionnaire as well as an IQ evaluation by means of the WAIS-III questionnaire to verify that they met the inclusion criteria. A week before acquiring the *f*MRI sequence, a second interview took place to describe the experimental procedure in detail, in addition to submitting the subjects to a training session in the completion of the tasks so that they would become familiar with the study before the *f*MRI session. No differences were detected at this point between groups in relation to the reaction time, number of correct responses or total errors. For the *f*MRI study, the subjects were asked to refrain from ingesting alcohol, tea, coffee, cigarettes or any other substance that might alter Central Nervous System (CNS) 12 hours prior to the study. Moreover, they were asked to attend with comfortable clothing and without any metallic objects. Minutes before the study, they were given the task instructions to read, and their blood glucose level was taken with a glucometer (AccuCheck Active).

### Data analysis

For the treatment of the images and the statistical analysis of the functional magnetic resonance imaging (*f*MRI), the software Statistical Parametric Mapping (SPM8) was used (http://www.fil.ion.ucl.ac.uk/spm/). All images were slice time and motion corrected, coregistered with a high-resolution anatomical scan, and normalized to Montreal Neurological Institute space. We conducted spatial smoothing using a Kernel Gaussian Full Width at half-Maximum (FWHM) of twice the size of the voxel. Additionally, the images were placed on a standard template using the above mentioned coordinates as an anatomical reference.

In relation to the influence of head motion, we estimated the root mean square and peak/average (across volumes) framewise displacement (FD), which is based on SPM’s realignment procedure. We used the same procedure described in [35] using the converted rotational displacements from degrees to millimeters by calculating displacement on the surface of a sphere. There were no statistically significant differences in any of these parameters for each group using a nonparametric Kolmogorov test.

A total of 124 brain volumes were obtained in each paradigm, and for experimental design reasons, 12 volumes were deleted per task, corresponding to the volumes generated at the times of the instructions, which were uninteresting for the study, thus yielding a total of 112 volumes per task in the statistical analysis.

The volumes corresponding to each task were analyzed separately. The statistical analysis of brain activation was conducted through a simple general linear model (GLM) using the correction for multiple comparisons of the family wise error rate (FWE) for an α level of 5%. All four analyses (one for each task and group) were conducted by simple contrasts between the BOLD signal means obtained during the activation periods and those obtained during the resting periods. For the analysis of the brain connectivity network, by means of the MarsBars 0.44 software, we extracted the activation data of the BOLD signal for each of the regions of interest (ROIs) that the previous statistical analysis had shown to be significantly activated during the task completion. For each region, we included in the reduction procedure, the total number of significant voxels in the first level analysis. The data matrices of the BOLD signal for each ROI were analyzed through the statistical analysis software M-PLUS 5.1, using a structural equation model (SEM) to extract the network of effective connectivity between the ROIs based on the matrix of correlations between ROIs.

## Results

### Behavioral results

The behavioral results are shown in more detail in our previous papers [32, 33]. In summary, the results showed that both groups had a high percentage of correct answers in tasks A and B (between 88.28% and 94.53% of correct answers). The only significant differences were observed in the interaction, tasks AB by Group [*F*(1, 30) = 4.35; *p* = .046; *η*^*2*^ = .133]. In particular, the patients showed a higher percentage of correct answers in task A and the controls in task B. In any case, the effect size of such difference was small, and the high percentage of correct answers for both groups in both tasks allowed us to confirm that our research was completely feasible in the BoxCar design we used and that the ROIs extracted in this analysis were activations related to the successful completion of the respective tasks.

### Activated areas

Table 2 show the list of activated areas during the completion of the different tasks separated by groups, controls and patients. As seen, there is a great number of areas activated in the frontal lobe, which is unsurprising since several frontal areas participate in WM. We should note that the largest ROIs are found in this lobe, with ROIs with over 14 clusters in some cases. This means that, although the point with the greatest activation is in the area shown in the table, there are ROIs comprising over 300 voxels with smaller activations in nearby areas. However, since they are activated at the same time and are located side by side, the neuroimaging analysis groups them into one. We can observe, in Table 2, which areas activated in the frontal lobe are on the right side of the brain. Among the most relevant areas activated in the frontal lobe, we find, in both tasks, the medial frontal gyrus, which is linked to high demand levels for executive functions.

**Table 2:** Activated areas for the different groups and tasks.

Group and Task	Num	H	Anatomical region	Broadmann Area	Coord T			Z stat
					X	Y	Z	
Control group in task A	1	R	Superior Frontal Gyrus	9	34	39	30	4.55*
	2	R	Middle Frontal Gyrus 6	6	28	1	47	6.12*
	3	R	Middel Frontal Gyrus 11	11	11	32	-17	4.89*
	4	R	Supramarginal Gyrus	40	50	-31	33	3.77*
	5	R	Declive		19	-75	-21	4.01*
	6	R	Superior Temporal Gyrus	9	34	39	30	6.11*
	7	R	Lentiform Nucleus Putamen		-28	-21	-4	3.91*
							
	8	L	Middle Occipital Gyrus	19	-45	-73	-14	4.12*
	9	L	Fastigium		-5	-53	-23	5.02*
	10	L	Culmen		-6	-45	-17	4.77*
	11	L	Declive		-6	-74	-21	3.99*
	12	L	Lentiform Nucleus Putamen		-28	-21	-4	6.12*
	13	L	Ventral Anterior Nucleus		-7	-3	3	5.12*
Patients group in Task A	1	R	Red Nucleus		8	-19	-10	3.44*
	2	R	Fusiform Gyrus	37	46	-52	-7	4.12*
	3	R	Inferior Temporal Gyrus	20	45	-20	-23	3.79*
							
	4	L	Red Nucleus		-5	-20	-9	3.88*
	5	L	Pyramis		-5	-69	-28	3.91*
	6	L	Declive		-6	-74	-21	4.22*
	7	L	Lingual Gyrus	18	30	-89	6	4.37*
Control group in Task B	1	R	Pulvinar		9	-20	8	4.11*
	2	R	Putamen		26	7	-4	3.79*
	3	R	Inferior Frontal Gyrus	9	34	39	30	4.52*
	4	R	Middle Frontal Gyrus	6	28	1	47	6.12*
	5	R	Superior Frontal Gyrus	8	22	28	42	5.11*
	6	R	Tuber		5	-74	-27	4.23*
	7	R	Lingual gyrus	18	30	-89	6	5.02*
	8	R	Subgyral Hippocampus		31	-39	5	3.81*
	9	R	Middle Temporal Gyrus	20	45	-20	-23	3.44*
							
	10	L	Precuneus	7	-18	-57	50	4.12*
	11	L	Precentral Gyrus	6	-28	0	48	5.11*
	12	L	Middle Occipital Gyrus	19	-45	-73	14	4.61*
	13	L	Uvula		-13	-49	-14	4.22*
	14	L	Supramarginal Gyrus	40	-52	-30	32	3.99*
	15	L	Declive		-6	-74	-21	4.91*
	16	L	Lentiform Nucleus		-28	-21	-4	3.95*
	17	L	Claustrum		-30	13	9	4.12*
	18	L	Pulvinar		-8	-18	8	4.38*
	19	L	Putamen		-28	-21	-4	5.23*
	20	L	Insula	13	-40	0	2	5.07*
Patients Group in Task B	1	R	Culmen		9	-33	-21	3.78*
	2	R	Fusiform Gyrus	37	46	-52	-7	4.44*
	3	R	Lingual Gyrus	18	30	-89	6	5.79*
	4	R	Declive		19	-75	-21	5.18*
							
	5	L	Cerebellar Tonsil		-17	-55	-41	3.82*
	6	L	Declive		-6	-74	-21	4.29*
	7	L	Red Nucleus		-5	-20	-9	4.51*
	8	L	Fusiform Gyrus	19	-45	-73	14	3.77*
	9	L	Culmen		-6	-45	-17	4.81*

*p < .001, H: Hemisphere; R:Right; L:Left; Z stat: Statistical Contrast; Coord T: Tailarach coordinates

### Effective connectivity network. SEM model estimation

The conception of the effective connectivity model though structural equations is based on the fact that the computation capacity makes it possible to identify cognitive processes as a complex series of hierarchically organized computational models. It is assumed that the processes analyzed are conceived as separable, and following the same logic as in cognitive subtraction, the final process is defined by the addition of defined partial processes.

By effective connectivity, we mean statistical models that formulate stochastic structural relationships between specific ROIs that have shown statistically significant activity with cognitive task.

The stages to estimate effective connectivity through SEMs were defined by [36]:

- Selecting ROIs through the combination of the univariate analysis, the intensity changes in the BOLD signal, the multivariate analysis, and the theoretical knowledge.
- Obtaining the anatomical model.
- Calculating interregional covariance or the matrix of correlations of the *f*MRI data.
- Calculating the trajectory of the coefficients and comparing them to the models according to the characteristics of the statistical estimation technique.

The SEMs applied in this environment are based on the so-called type III models, by [37] and are identified by the following general expression:

$$y_{t}=\beta y_{t}+\zeta_{t},$$

where *y*_*t*_ are the values of the ROIs selected, *β* the parameter estimations, and *ζ*_*t*_ the structural errors associated with each endogenous variable *y*_*t*_.

We need to bear in mind two important considerations. The first consideration regards the concept of effective connectivity: SEM estimation is generated regardless of the biological structure of the nervous system. The effects described deriving from the univariate and multivariate analyses lack neurofunctional support and are solely justified by statistical effects which must later be contrasted with the existing literature.

The second consideration is statistical. Whatever the parameter estimation technique—in our case Maximum Likelihood—it involves estimating the parameters of matrix *β*, which comprises the effects between ROIs, and the parameters of matrix *ψ*, which comprises the variance-covariance matrix between the structural errors *ζ*_*t*_, so that *ψ = E(ζζ’)*. The specific form adopted by *β* derives from the effects specified between ROIs, while the form adopted by *ψ* summarizes the assumptions specified regarding the structural error distributions. Generally, the classical assumptions of SEMs would involve initially assuming that *E(ζζ’) = E(y*_*t*_*ζ’)* = 0 and, consequently, the errors should be uncorrelated between themselves with regard to the endogenous variables, except for the possibility that matrix *β* considers nonrecursive effects.

Evidently, this enables the possibility that the error distribution may be independent from the estimations of *β*. In fact, this model also implies the assumption that the variables—that is, the values for each ROI—are observed continuous variables of multinormal distribution. The truth is that each *y*_*t*_ value representing the *f*MRI activity of one ROI is estimated by a component principal analysis based on the selection of a given number of voxels convoluting under a geometric form defined around a voxel of maximum univariable or multivariable statistical significance under the statistical assumptions of the massive general linear model. Therefore, each *y*_*t*_ extracted might be considered a latent variable (*η*_*t*_) defined on the basis of the values actually observed in every voxel and estimated according to the type of design used, block design or event-related.

However, despite certain limitations of the model, SEM complex models are essential for the study of connectivity as it would be imprudent not to assume the existence of reciprocal effects between ROIs. In addition, the goodness-of-fit indicators, the Tucker and Lewis Index and the Determination Coefficient (*R*^*2*^), indicate that the models for each task and group fit perfectly. To establish the best structural model in each of the four variance and covariance matrices derived from the ROIs of each group and task, we followed the procedure described by [34]. It involves starting the estimation of all the possible models and discarding all those incorrectly specified (degrees of freedom below 0); discarding those models that do not converge after a reasonable number of steps (500 iterations in this case); and finally, out of the models that pass these previous stages, selecting the one with the best fits. These are the models resulting from connectivity studies.

#### Results task A

The effective connectivity model of the control group in task A shows major direct interconnections between the frontal lobe and sublobar regions, such as the right anterior ventral nucleus. In turn, the latter sends outputs to the cerebellum (declive) by connecting with the putamen and the occipital lobe. Additionally, Brodman’s area 6 of the medial frontal gyrus is indirectly connected with the anterior ventral nucleus by means of the superior temporal gyrus and passing through the supramarginal gyrus. The fastigium and the culmen, although anatomically identified in another lobe, are also part of the cerebellum and are in charge of sending outputs to the frontal lobe, the declive, and the parietal lobe.

**Fig 2:**
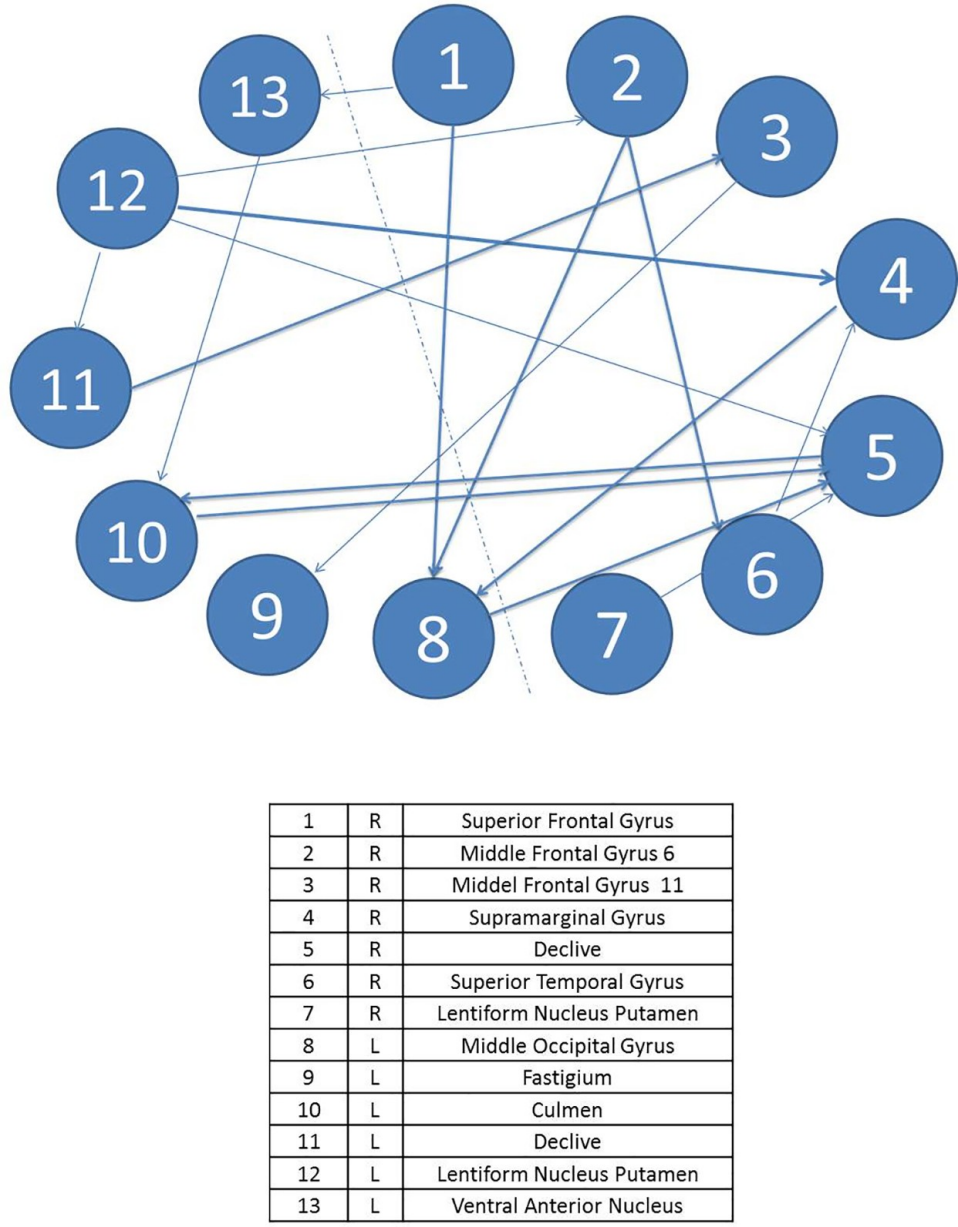
Path diagram and results of the SEM of task A for patients. The grey line represents left and right hemisphere. Note: TLI = Tucker Lewis Index; CFI = Comparative Fit Index; AIC = Akaike Information Criteria; BIC = Bayesian Information Criteria. R2 = Coefficient of Determination; χ2 = Goodness-of-fit statistic.

**Fig 3:**
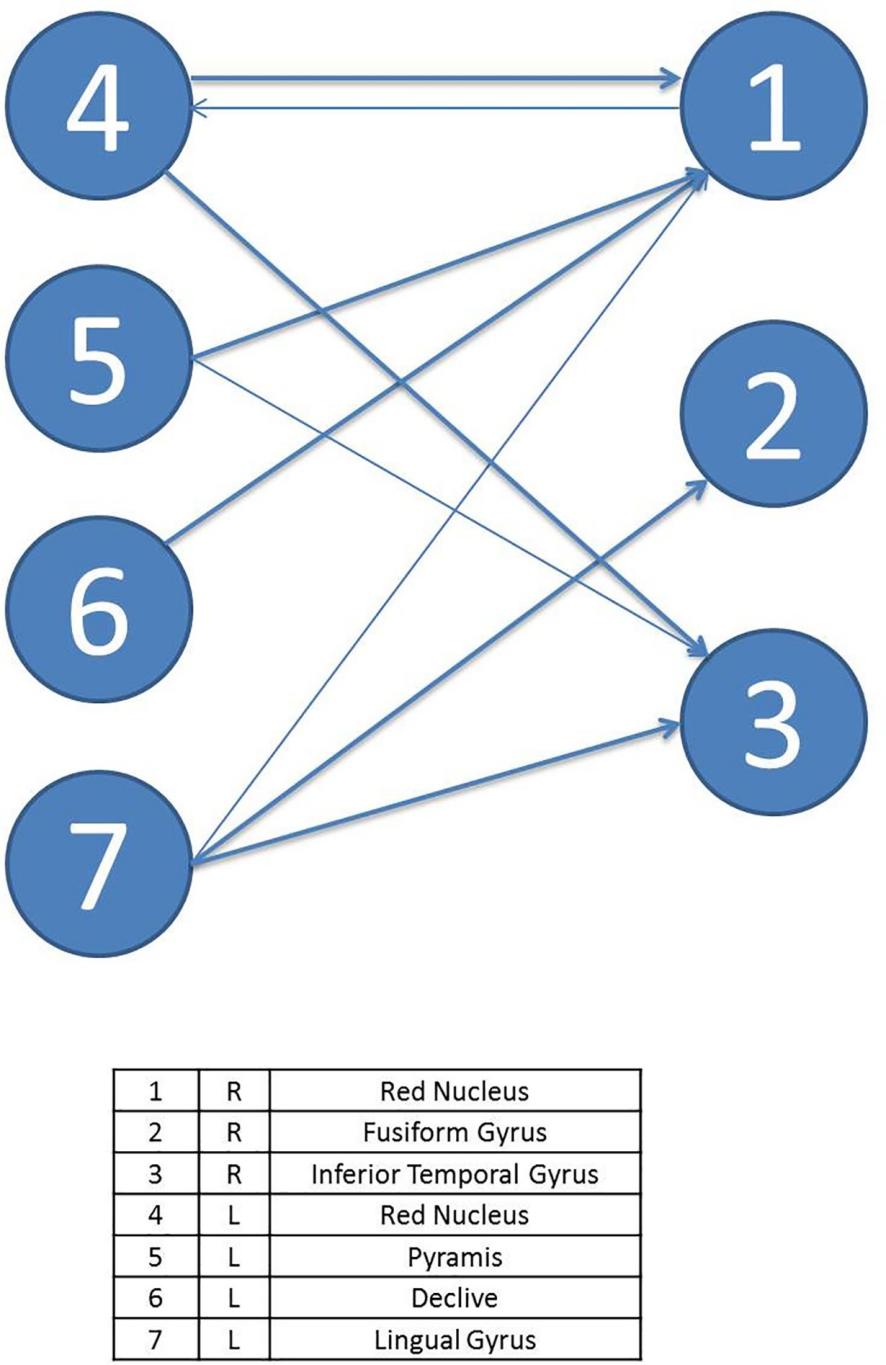
Path diagram and results of the SEM of task A for controls. The grey line represents left and right hemisphere. Note: TLI = Tucker Lewis Index; CFI = Comparative Fit Index; AIC = Akaike Information Criteria; BIC = Bayesian Information Criteria. R2 = Coefficient of Determination; χ2 = Goodness-of-fit statistic.

The effective brain connectivity network modeling for patients starts off with a lower number of ROIs deriving from the univariate and multivariate analyses of the changes in intensity of the BOLD signal. In turn, the areas appearing as significantly activated are more related to the motor completion of the task than to the executive functions network—such as the red nucleus, responsible for motor coordination—and have a lower number of connections between themselves. Figs 2 and 3 display the diagrams for each complex system by means of SEMs as well as the main statistical estimations. In relation to individual parameter estimation, in the control group, there are 13 ROIs connected with a total of 17 significant paths (*β*_*ij*_ in terms of SEM nomenclature); meanwhile, in the T1D group, there are only 7 ROIs connected with 8 significant paths.

#### Results Task B

The effective connectivity network model in the control group during the completion of task B is the most complex of all. This is due to the increased difficulty of this task. Lateralization can be clearly observed in the direction of the connections from left to right, especially from sublobar areas such as the thalamus, the putamen, and the claustrum towards areas of the anterior lobe, the hippocampus, and the temporal lobe. The hippocampus is also the area receiving the most connections from different lobes, as this is an organ involved in long-term memory and essential for spatial navigation.

**Fig 4:**
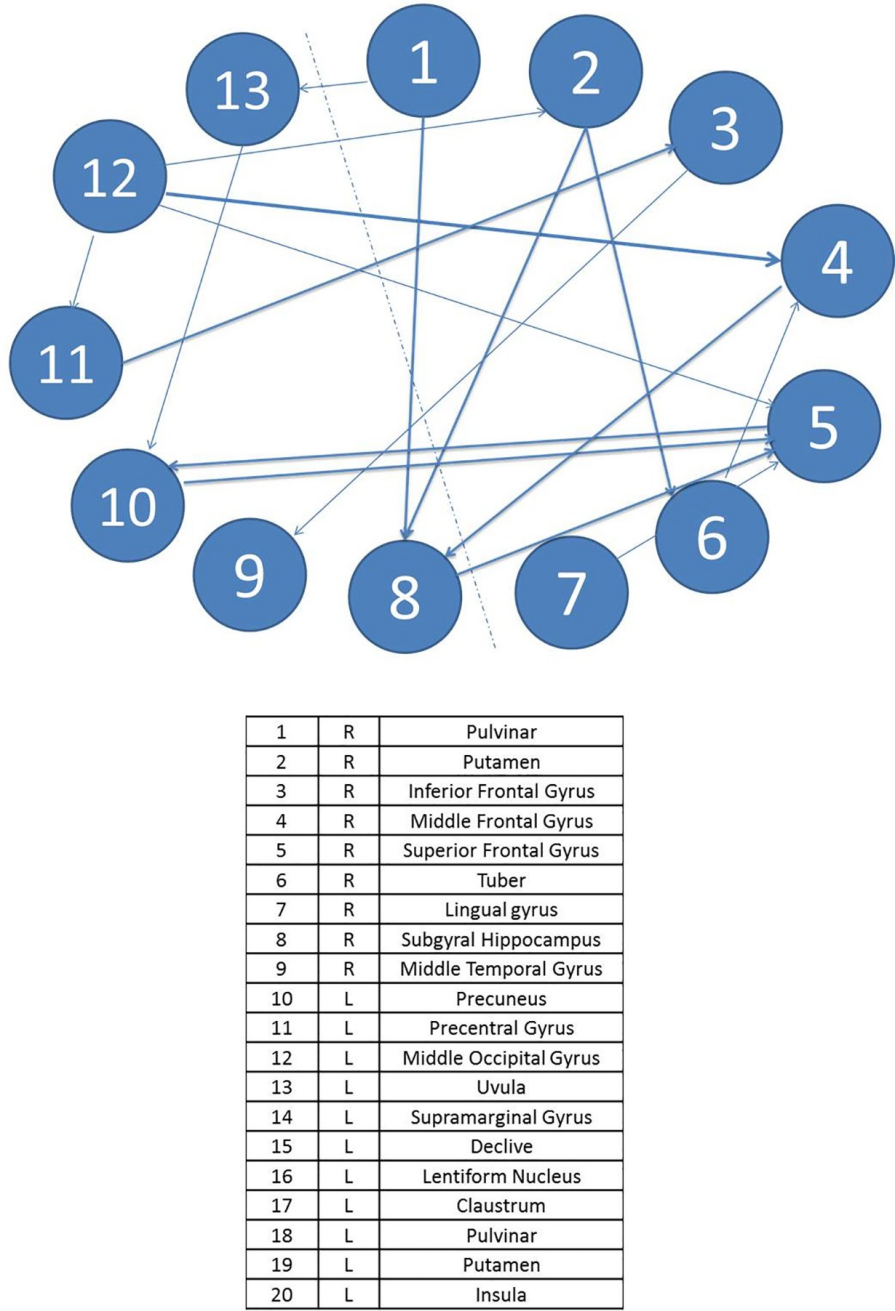
Path diagram and results of the SEM of task B for patients. The grey line represents left and right hemisphere. Note: TLI = Tucker Lewis Index; CFI = Comparative Fit Index; AIC = Akaike Information Criteria; BIC = Bayesian Information Criteria. R2 = Coefficient of Determination; χ2 = Goodness-of-fit statistic.

**Fig 5:**
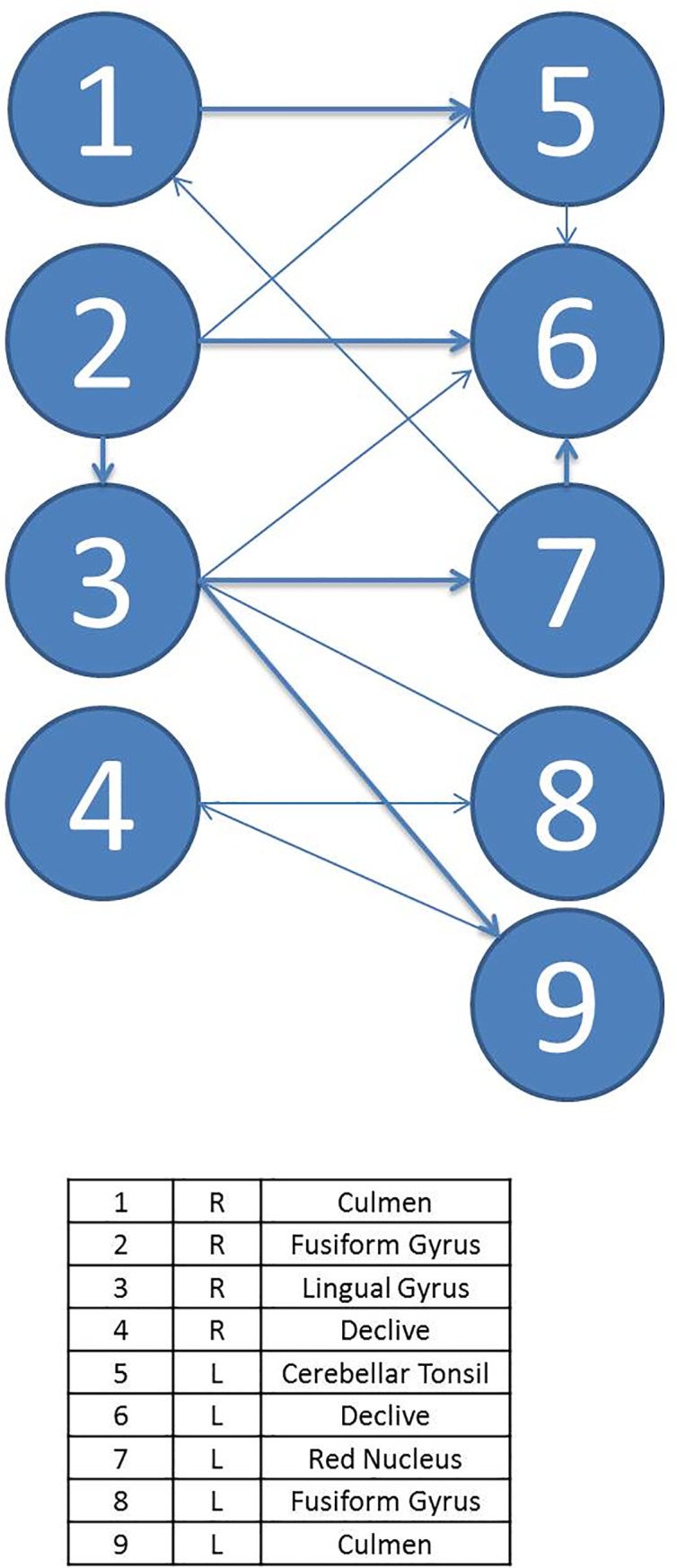
Path diagram and results of the SEM of task B for controls. The grey line represents left and right hemisphere. Note: TLI = Tucker Lewis Index; CFI = Comparative Fit Index; AIC = Akaike Information Criteria; BIC = Bayesian Information Criteria. R2 = Coefficient of Determination; χ2 = Goodness-of-fit statistic.

As in task A, patients show fewer activated regions in task B. In this case, no activation is shown in the main areas of executive functions or in any area of the frontal lobe. However, the culmen of the anterior lobe, the red nucleus, and the declive do appear. Figs 4 and 5 displays the diagrams for each complex system by means of SEMs as well as the main statistical estimations. In this case, in the control group, there are 20 ROIs connected with a total of 18 significant paths (*β*_ij_ in terms of SEM nomenclature); meanwhile, in the T1D group, there are only 9 ROIs connected with 13 significant paths.

Additionally, we obtained the global fit values for every model, and they guaranteed that each estimated model was the best possible model out of all the models analyzed according to [34] procedure. Table 3 shows these values. Finally, we summarized, in Table 4, the values of the standardized structural parameters to offer the empirical values of the different effects, both for those with a significance p < .01 (thin line in the figures) and for those with a significance p < .001 (thick line in the graphs).

**Table 3:** Global fit index for each structural model.

Model	TLI	CFI	AIC	BIC	R^2^	χ^2^	p
Task A Group Control	.99	.99	-47234.89	-48231.12	.94	3211.51	.845
Task A Group Patients	.99	.99	-234561.12	-277152.33	.92	2213.11	.781
Task B Group Control	.99	.99	-88237,11	-89122.37	.95	1221.11	.712
Task B Group Patients	.99	.99	-416231.31	-418321.77	.92	877.23	.621

TLI = Tucker Lewis Index; CFI = Comparative Fit Index; AIC = Akaike Information Criteri; BIC = Bayesian Information Criteria; R^2^: Coefficient of Determination; χ^2^ = Chi-Square estimation; p = p value associated to χ^2^.

**Table 4:** Estimation of the free parameters for each model differentiated by intensities.

Model	Line	ML Parameter estimation
Task A Group Control	Thin	*β*_*ij*_ = .213 to .301 p < .01
	Thick	*β*_*ij*_ = .377 to .471 p < .001
Task A Group Patients	Thin	*β*_*ij*_ = .213 to .301 p < .01
	Thick	*β*_*ij*_ = .377 to .471 p < .001
Task B Group Control	Thin	*β*_*ij*_ = .178 to .278 p < .01
	Thick	*β*_*ij*_ = .310 to .418 p < .001
Task B Group Patients	Thin	*β*_*ij*_ = .178 to .278 p < .01
	Thick	*β*_*ij*_ = .310 to .418 p < .001

ML: Maximum Likelihood estimation

## Discussion

In this paper, we have studied, through SEMs, the connectivity of two visuospatial memory tasks: one that we might consider short-term memory and another with a greater WM component. Some authors consider the distinction between both types of memory to reflect the degree of manipulation, and the distinction between both types of memory would accordingly be rather gradual than categorical. Consequently, the term ‘working memory’ might be applied to immediate memory tasks [15]. Both tasks used in this study shared a good deal of the cognitive processes that were involved. However, Task B also has a rehearsal component of the information that is unnecessary to solve task A. Therefore, it is hardly surprising that, to a certain extent, the ROIs activated and some aspects of effective connectivity were similar in both tasks.

### Control group

The control group–in task A—showed an effective connectivity pattern comprising numerous brain areas. First, we should point out two classical ROIs in these tasks: the right superior frontal circumvolution and the right middle frontal circumvolution (BA 6 and 11). The superior frontal circumvolution is a brain area classically involved in executive control and involved in the updating of memory content and the storage of temporal stimuli order [16], which are two key cognitive components in this type of task. It is a point of convergence for the dorsal and ventral networks of the attention network, and it is responsible for slowing down endogenous attentional processes and directing attention towards external stimuli. Moreover, this cortical area is linked to processes related to decision-making [38, 39].

Other areas that appear activated as ROIs and generate effects on the connectivity model are the cerebellum fastigium, the declive, and the culmen. The declive would be an area constantly related to WM [15]. The cerebellum nuclei activated in our research were found in the left hemisphere, and they send most of their effects to the cerebellum’s right hemisphere and the right middle and superior frontal cortexes. This interaction between the left cerebellum and the right prefrontal cortex is to be expected due to the demonstrated cerebellar role in the timing [40] and sequencing of stimuli in WM tasks [41, 42].

The left and right putamen also generated and received effects in this task. Some studies suggest that the left putamen would be a brain area involved in preventing irrelevant information from entering WM [43]. Undoubtedly in our study, the area receiving the most effects is the thalamus’s anterior ventral nucleus. It has been purported that in WM memory tasks, the thalamus participates in holding in memory and retrieval phases [44, 45].

However, none of these papers refer specifically to the thalamus’s lateral nuclei or the anterior ventral nucleus, since the role of the lateral thalamus in spatial memory is little known [20]. Finally, we also observed activations in the supramarginal parietal cortex in this group. This is a somewhat surprising result, as it has usually been observed that the storage of spatial material tends to activate the superior parietal cortex (BA 7), while the storage of two objects or forms is what activates the inferior parietal cortex [15, 16].

In task B, in addition to the activations observed in task A that we discussed above, in this connectivity model we saw activations in the inferior frontal cortex, a brain area that would be important for storing visuospatial material in WM tasks and related to the manipulation of the content in WM [16]. Task B in our study had a rehearsal component that was unnecessary to solve task A. Another ROI activated in task B for this group was the insula. This is a structure that is consistently activated in WM paradigms [15] and related to choosing correctly in WM tasks [46]. Another area that is part of the model is the precuneus, which is classically involved in the spatial or verbal storage of information in this type of task [16]. Additionally, we detected activations in the middle occipital gyrus, an area that has been related in the storage of short-term visual memory [47]. We must point out that in this task, the brain area receiving the most effects in the estimation of connectivity is the R-hippocampus. This brain area has classically been related to long-term memory, but more recent studies suggest that the different subregions of the medial temporal lobe may be critical for the short-term storage and manipulation of complex spatial arrangements in WM tasks [21].

### T1D group

In the group of T1D patients, the connectivity model for tasks A and B showed a very similar pattern, with a smaller number of brain areas involved, and there is a remarkable lack of frontal cortical areas. However, another remarkable aspect was the fact that in task A, the region receiving the most effects was the red nucleus, which receives its projections mainly from the cerebellum. In task B, important connectivity effects appear in the model that are related to the red nucleus, although this time is only the left side, as it shows connectivity with some cerebellum nuclei. The red nucleus (RN) has been classically considered a motor nucleus with important direct pathways with the dentate nucleus in the cerebellum [48]. Structural data obtained with diffusion–tensor imaging suggested that the red nucleus is connected with the thalamus, the basal ganglia and is also connected directly and indirectly with the association cerebral cortex. Thus, corticorubral pathways may directly connect the red nucleus with the motor, premotor several areas of the superior, middle and dorsolateral prefrontal cortex insula, and temporal and cingulate cortices. Indirectly, through cerebellar pathways, the red nucleus is connected both ipsilaterally and contralaterally to prefrontal, parietal and occipital cortices [49, 50]. Functional neuroimaging studies suggest that the RN is functionally connected to brain areas related to WM, including prefrontal cortical areas (BA 45; BA 46; BA 47), the insula, the hippocampus (BA 11 and precuneus) and the occipital cortex [51]. In addition, a recent study [52] using high resolution PET showed very similar results to the study [51] and suggested that the right RN showed strong ipsilateral metabolic correlations with association cortices, while the left RN showed a more bilateral pattern of connectivity with the cerebral cortex [52]. These findings suggest that the RN shows strong connectivity with cortical areas that are either part of the “core” WM network [15] or had shown activity during visuospatial WM tasks, and several of them showed activity and connectivity effects in our healthy sample. The most significant effects towards the RN in our study came from the cerebellum pyramid and declive. Some studies consider the cerebellar tonsil and pyramid to be involved in the executive control of WM by preventing irrelevant information from entering WM [53], and the meta-analysis by [15] suggested that the declive would be an area consistently involved in this cognitive function. In addition, another study examining cerebellar connectivity suggests that the cerebellum contributes to the salience network, the DMN, and the cognitive control network, and their results also showed that the RN also participates in those three networks [48]. Another area receiving important effects in both tasks is the bilateral lingual circumvolution, as does the right fusiform circumvolution. It has been reported that both could be activated in healthy persons in WM tasks for forms but not in spatial location tasks [54]. Other studies suggested that the left lingual circumvolution can be activated in visuospatial WM tasks [21].

Taken together, our data suggested that the T1D patients showed a connectivity pattern during our memory task that is quite different from the one shown by healthy controls, implicating the RN, the cerebellum, and the lingual and fusiform cortices. This will be interpreted as a compensatory mechanism that patients need to efficiently solve the WM tasks but that relays to encephalic structures that are also related to WM. We should note the absence of the different areas of the prefrontal cortex in the model, but in our previous studies, the T1D patients presented fewer activations than the control group [32, 33]. This cerebellar-RN connectivity pattern during the WM task was previously found by [55] in patients with attention-deficit hyperactivity disorder. Our data, along with the findings of those authors, suggest that when faced with pathologies that affect the brain from an early age, such as ADHD and T1D, the brain might develop this adaptation that would be relatively specific for WM. We must, however, be very cautious when making this interpretation because we did not have this starting hypothesis in our study, and our results are derived from a data-driven analysis.

Be that as it may, our study has some limitations that need to be addressed. We based the preparation of our effective connectivity models on a data-driven strategy, which brings about complex connectivity models with numerous brain areas involved. This is especially true in the group of control participants. For that reason, the conclusions obtained from this study and the way they are interpreted must necessarily be taken with caution and as a preliminary approach, which leads to future papers driven by more specific hypotheses and approaches to connectivity with seed studies. Nevertheless, it should be clear that our main goal was to study connectivity in visuospatial WM in patients with T1D. Given that it had never been done before and that the data consistently show that diabetic patients have brain activation patterns clearly different from those of healthy persons in this type of task [23, 32, 33], an approach of this kind is the most suitable. In this sense, it should be considered that our data confirm *a posteriori* the idea that this is a sensible approach to the study of the phenomenon if we bear in mind that the effective connectivity pattern in the group of T1D in both tasks is very different from that of the control participants.

Another limitation of the study is that it should be understood and interpreted in the context of the tasks used. An aspect hindering the study of *f*MRI brain databases is the existence of significant heterogeneity of WM tasks, with a great variety of stimuli, and other manipulations, such as memory load, delay times, and the presence of distractors, among others [15]. All these variables exert great effects on the activation of several brain areas [15, 16]. Consequently, our SEM connectivity models in WM must be understood in the context of the tasks used in the study, and further studies should be conducted with other tasks to support or refute the finding of rubro-cerebellar connectivity in the group of T1D. Be that as it may, this is a limitation that we will find in virtually any brain connectivity study that uses an SEM connectivity estimation approach.

Likewise, our data have some strong points that we must highlight. First, this is the first paper to study brain connectivity in WM tasks in patients with T1D using an SEM model. The data we obtained as a whole suggest that these patients develop a series of functional brain adaptations that would have a preventive or limiting effect on the alterations of this cognitive function. This is not new, as several papers have reached similar conclusions [23, 28, 32, 33]. However, our data suggest that these adaptations might be very important, as patients with T1D develop effective connectivity networks in visuospatial WM that are very different from those of healthy persons. This is an unquestionable contribution of our study, given that classical *f*MRI studies do not show such quite differentiated brain activity patterns between patients with T1D and control participants. Second, the estimation of effective brain connectivity in this study was conducted on the basis of a BoxCar design, which, according to certain studies, would be a more sensitive design to estimate connectivity with SEM than event-related designs or studies at rest [26]. We have also analyzed a considerable number of brain areas. Although this is an added difficulty to the analysis, it is also closer to the brain’s complexity and to the cognitive function studied than what might be seen in many SEM models published today [26]. In this sense, we must point out that WM is altered in almost any neurological or psychiatric disease, which indirectly proves that numerous brain areas take part in this cognitive function [15].

Another strong point of our study is the fact that, despite its exploratory nature, the fit of the SEM models in both tasks and groups is very good and has a very high proportion of explained variance in all the models. In this sense, we must comment on the fact that our sample comprises 15 participants in each group, which is adequate from the point of view of sample size for *f*MRI studies [24]. We must emphasize that when preparing the model, we conducted a thorough review of the conditions of application of the SEM model, paying close attention to the compliance with the model’s assumptions and the distribution of each of the ROIs. All of the results lead us to consider that the conclusions we have obtained in the current study are reasonable despite the study’s exploratory nature. Further studies are necessary in combination with other approaches to the data analysis (e.g., seed studies) to confirm the role of the different brain structures, both subcortical and cerebellar, in WM T1D patients and to establish more firmly the connectivity networks associated with this cognitive function in this type of patient.
